# One-Pot Synthesis of Pt–Fe for EOR: Effect of pH

**DOI:** 10.1021/acsomega.6c09204

**Published:** 2026-10-19

**Authors:** Marco Antonio Di Francia, Maria Portarapillo, Antonella Gianna Tesone, Almerinda Di Benedetto, Giuseppina Luciani

**Affiliations:** Department of Chemical, Materials and Production Engineering, 9307 University of Naples Federico II, P.le Tecchio 80, Naples 80125, Italy

## Abstract

The development of efficient electrocatalysts for the ethanol oxidation reaction (EOR) remains a major challenge for direct ethanol fuel cells (DEFCs) due to slow kinetics and catalyst poisoning by reaction intermediates. Herein, Pt/C and Pt–Fe/C catalysts were prepared via a one-pot polyol synthesis, and the effect of pH on Fe incorporation and catalytic performance was investigated. X-ray diffraction showed lattice contraction in Pt–Fe catalysts, confirming Fe incorporation, which was more pronounced under alkaline conditions. Electrochemical measurements demonstrated a strong dependence on both Fe addition and pH. Pt–Fe/C at pH 11.4 achieved a mass activity density of 850 A/g_Pt_, outperforming not only Pt–Fe/C neutral and Pt/C but also the commercial Pt/C catalyst at 20 mV/s. Moreover, a lower onset potential (0.479 V) and a higher If/Ib ratio (1.9) indicate faster reaction kinetics and qualitatively suggest tolerance to adsorbed intermediates. The enhanced performance is attributed to the synergistic effect of Fe incorporation through electronic and bifunctional mechanisms.

## Graphical abstract

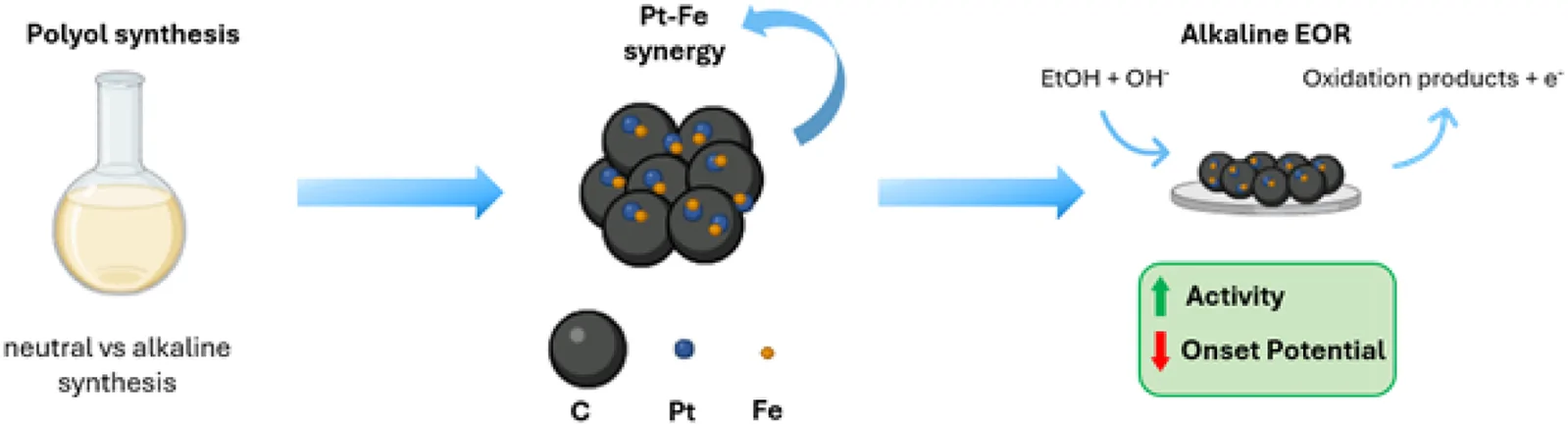

## 1 Introduction

The increasing global demand for clean and sustainable energy has intensified research on efficient electrochemical energy conversion technologies. Among these, direct alcohol fuel cells (DAFCs) have gained considerable attention due to their high energy density, ease of fuel storage and transport, and suitability for both portable and stationary applications.^1^ In particular, direct ethanol fuel cells (DEFCs) have attracted increasing attention because ethanol can be produced from renewable sources and combines low toxicity and low volatility with a high energy density.^2,3^

Despite these advantages, the widespread application of DEFCs is still limited by the slow kinetics of the ethanol oxidation reaction (EOR).^4^ The EOR is a complex multistep process involving ethanol adsorption, successive dehydrogenations, C–C bond cleavage steps, and the formation of a variety of reaction intermediates and products. Two main reaction pathways are typically identified: the complete oxidation pathway (C1), leading to CO_2_, and the partial oxidation pathway (C2), leading to acetic acid and/or acetaldehyde.^5^ On conventional platinum-based catalysts, the reaction predominantly proceeds via the C2 pathway, resulting in incomplete oxidation and reduced energy efficiency.^5–7^ Moreover, a major limitation of Pt catalysts lies in their susceptibility to poisoning by strongly adsorbed intermediates, such as CO-like species formed during ethanol oxidation. These intermediates occupy active sites and inhibit further reaction, significantly reducing catalytic activity and long-term stability.^5^ Therefore, improving the poisoning tolerance and facilitating the oxidative removal of reaction intermediates remain critical objectives in the development of efficient EOR electrocatalysts.

To overcome these limitations, significant efforts have focused on modifying Pt catalysts through alloying with secondary metals. The addition of transition metals such as Ru,^8,9^ Sn,^10^ Ni,^11^ Co,^12^ and Fe^13^ has been widely reported to improve catalytic performance via two main mechanisms: the bifunctional effect and the electronic ligand/strain effect.^14^ Among these, iron has recently attracted growing interest due to its low cost, natural abundance, and favorable electronic properties.^15^ Incorporating Fe into Pt-based structures can alter their electronic structure, weakening the adsorption of poisoning species and improving the catalyst tolerance. In addition, owing to its oxyphilic character, Fe-containing sites can also promote the formation of additional hydroxyl species, which facilitate the oxidation and removal of CO and other intermediates from adjacent Pt sites, in accordance with the bifunctional mechanism.^16^ The final catalytic properties of Pt–Fe materials, however, strongly depend on the synthesis route, which can affect the composition, particle size, dispersion, and, eventually, alloy formation.

In the literature, several methods have been proposed for synthesizing electrocatalysts. Typical nanoparticle synthesis methods include wet-chemical,^17^ solvothermal/hydrothermal,^18,19^ deposition methods^20^ and solid-state thermal methods.^21^ In particular, the wet-chemical route features mild conditions such as temperature and pressure and is easy to set up and scale up.^22^ In this category, the polyol method is emerging among others due to its further advantages, such as high control over size and shape. The slightly high temperature (100–320 °C) enhances the chance of obtaining well-crystallized materials.^23^ Moreover, this method enables the easy formation of metallic nanoalloys and in some cases ordered intermetallics without any subsequent thermal treatment.^24^ Additionally, polyols are biocompatible, biodegradable, and less toxic than other solvents and reducing agents. Hence, polyols are classified as green solvents, making them interesting for industrial-scale nanoparticle synthesis.^23^ For these reasons, the polyol process has emerged as a versatile and widely used method for the synthesis of metal nanoparticles with controlled size, morphology, and composition. In this approach, polyols such as ethylene glycol act both as a solvent and a reducing agent, enabling the formation of metallic nanoparticles under relatively mild conditions.^25^ When combined with suitable supports, this method offers several advantages, including strong metal–support interactions, typically resulting from the in situ formation of nanoparticles, as well as a high surface area and mesoporous morphology, which are beneficial for mass transport and catalytic activity. These systems often exhibit improved water management, increased resistance to corrosion in acidic environments, and easier catalyst recovery.^22^ Moreover, one of the key advantages of the polyol method is the possibility of performing one-pot syntheses, thereby avoiding multistep procedures and facilitating the direct formation of alloyed nanostructures. Finally, the method allows for the precise adjustment of synthesis parameters, such as temperature, precursor concentration, and pH, which can have a significant influence on the nucleation, growth, and reduction pathways of metals.^22^

Among these parameters, the pH of the reaction medium plays an important role in determining the properties of Pt-based catalysts. It directly affects both the reduction kinetics of Pt precursors and their interactions with the carbon support. Under alkaline conditions, glycolate ions form and act as stabilizing agents, coordinating Pt species and controlling nucleation and growth, which typically leads to smaller and more uniformly distributed nanoparticles. At the same time, increasing synthesis pH can also increase the negative surface charge of both Pt colloids and carbon supports, creating electrostatic repulsion that may hinder metal deposition. As a result, while alkaline conditions improve particle dispersion and size control, they may also reduce Pt loading and influence the efficiency of alloy formation with secondary metals.^26^ Despite the growing interest in Pt–Fe electrocatalysts, the effect of OH^–^ concentration during polyol synthesis on Fe incorporation, the resulting structural properties, and EOR performance have not yet been systematically clarified.

Beyond the active metal phase, the choice of the support material is equally important for overall catalyst performance. An ideal support should provide high surface area for nanoparticle dispersion, strong metal–support interaction, good electrical conductivity, and stability under electrochemical conditions.^27^ Carbon-based materials, such as Vulcan XC-72R, are widely used because of their high surface area, good conductivity, and relatively low cost. Moreover, the polyol synthesis method promotes strong interaction between metal nanoparticles and carbon supports, leading to improved dispersion and enhanced catalyst stability.^22^

In this study, Pt/C and Pt–Fe/C catalysts were produced through the polyol method under natural and alkaline conditions in order to systematically investigate the effect of synthesis pH on Fe incorporation, on catalyst structure, and on the resulting electrocatalytic performance toward ethanol oxidation. While Pt–Fe catalysts and polyol synthesis have been widely investigated, the specific influence of OH^–^ concentration on the formation of Pt–Fe alloys and on the resulting EOR behavior has not yet been systematically clarified. Here, the effect of synthesis pH is examined through a direct comparison of monometallic and bimetallic catalysts prepared under identical conditions, combining structural, compositional, and electrochemical characterization. Structural characterization was carried out using X-ray diffraction (XRD), UV–vis spectroscopy, and SEM-EDX to evaluate Fe incorporation into the Pt lattice. Electrochemical techniques, including cyclic voltammetry, linear sweep voltammetry, chronoamperometry, and EIS, were employed to assess the catalytic performance and to examine the effect of pH in comparison with Pt/C. In addition, local pH variations during EOR were probed using HER as an electrochemical pH indicator, providing further insight into the role of Fe incorporation in modifying the interfacial hydroxide environment.

## 2 Materials and Methods

### 2.1 Materials

Platinum­(II) acetylacetonate, iron­(II) chloride tetrahydrate, ethylene glycol, KOH pellets, and perchloric acid (HClO_4_) 70% were purchased from Sigma-Aldrich, St. Louis, MO, USA, and utilized at reagent-grade. Nafion dispersion 10% D10 was purchased from Fuel Cell Store, Bryan, TX, USA. Vulcan XC-72R was purchased from BC Berlin Catalyst GmbH, Berlin, Germany.

### 2.2 Catalyst Synthesis

The syntheses of platinum (Pt) and platinum–iron (Pt–Fe) alloy nanoparticles were performed using the polyol method. In brief, a solution of 40 mL of water and ethylene glycol (1:3 vol.) was prepared in a round-bottom, three-neck flask. To evaluate the effect of the solution pH, the required samples were adjusted to a pH of 11.4 with KOH pellets to 11.4 (OH^–^/Pt ratio equal to 40).^26^ Then, 41.7 mg of Vulcan Carbon (VC) was added, and the dispersion was sonicated for 10 min. Additionally, platinum­(II) acetylacetonate and iron­(II) chloride tetrahydrate were added, giving a total concentration of 2 mM with a Pt:Fe ratio of 7:2.^28^ The system was then placed under reflux for 5 h at 160 °C and 250 rpm under an inert atmosphere. For comparison, a catalyst containing only Pt was prepared in the same conditions. After the reaction time, the system was allowed to cool down naturally, then vacuum-filtered using a nylon membrane with a pore diameter of 0.47, and washed with distilled water. The solid was then vacuum-dried at 60 °C and then collected from the membrane. Four samples were prepared: Pt/C neutral, Pt/C pH 11.4, Pt–Fe/C neutral, and Pt–Fe/C pH 11.4.

### 2.3 Physiochemical Characterization

The prepared catalysts were characterized by X-ray diffraction (XRD) with a Malvern PANalytical diffractometer (Malvern, UK) with a nickel filter and a Cu Kα radiation source (λ_*K*α*1*_ = 1.54060 Å) to determine the metal phases present. The average crystal size (τ) is calculated using the Scherrer formula:

1 $$\tau =\frac{K\cdot \lambda }{\beta \cdot \cos\theta }$$

where τ is the mean size of the crystallite sites, *K* is the shape factor, λ is the X-ray wavelength, β is the full width at half-maximum (fwhm), and θ is the Bragg angle. Analogously, the *d*-spacing (*d*) and lattice parameter (*a*) are calculated by the Bragg formula:

2 $$d=\frac{\lambda }{2\cdot \sin\theta }$$

3 $$a=d\cdot \sqrt{h^{2}+k^{2}+l^{2}}$$

where *h*, *k,* and *l* denote the Miller indices of the crystallographic planes.

To quantify the platinum content in the catalyst, the filtered supernatant was analyzed using a Jasco-V560 UV–visible spectrophotometer in a quartz cuvette at 253 nm. The obtained absorbance was used to calculate the concentration (*c*) using an absorbance–concentration calibration curve obtained, the method for which is described elsewhere.^29^ Therefore, the platinum loading is calculated as follows:

4 $$\%Pt_{cat}=\frac{\left(c^{0}-c^{\prime }\right)\cdot V_{sol}}{m_{cat}}\cdot 100$$

where *c°* and *c′* are the concentrations of Pt in the solution before and after the reaction in mg/L, *V*_*sol*_ is the volume of the reaction in liter, and *m*_*cat*_ is the mass of the obtained catalyst expressed in mg.

Metal content was quantitatively determined by inductively coupled plasma mass spectrometry (ICP-MS) using an Agilent 7500 ICP-MS instrument, after dissolution of the catalysts in nitric acid solution.

SEM-EDX was performed to assess the morphology and metal distribution of the samples with a TESCAN CLARA UHR SEM (Brno, Czech Republic) for morphology assessment paired with Oxford EDS (Abingdon, UK).

### 2.4 Electrochemical Setup and Characterization

The catalyst inks were prepared by dispersing 2 mg of catalyst in 1 mL of solution of water-isopropanol (4:1 vol.) and sonicated for 30 min with ice. Subsequently, 2 μL of Nafion D10 dispersion was added in the dispersion and sonicated for further 30 min with ice. Then, an aliquot of 18.4 μL of the ink was deposited onto a glassy carbon disk electrode (5 mm diameter), allowed to dry at room temperature with a rotation of 500 rpm and used as the working electrode. Measurements were conducted in a three-electrode cell using Autolab PGSTAT204 (Metrohm) with an FRA32M module to conduct Electrochemical Impedance Spectroscopy (EIS). The counter and reference electrodes were a platinum wire and a saturated Ag/AgCl electrode. All the results are reported by converting the measured potential to the Reversible Hydrogen Electrode (RHE) according to the following formula:

5 $$E_{RHE}=E_{Ag/AgCl}+{E^{{}^{\circ}}}_{Ag/AgCl}+0.059\times pH$$

where E_Ag/AgCl_ is the measured potential, ${E^{{}^{\circ}}}_{Ag/AgCl}$ is the standard potential (+0.222 V), and pH is the pH of the electrolyte solution.

Specifically, cyclic voltammetry (CV) was performed in 0.1 M HClO_4_ and nitrogen saturated atmosphere. The catalysts were first activated by cycling from 0.00 to 1.00 V vs RHE at a scan rate of 0.2 V/s 50 times. Voltammograms were obtained by cycling from 0.00 to 1.00 V vs RHE at 0.2, 0.1, 0.05, 0.02, and 0.005 V/s for 3 times each. The electrochemical surface area (ECSA) is calculated in the Pt hydrogen desorption region of the 0.05 V/s scan,

6 $$ECSA\left(\frac{m^{2}}{g}\right)=\frac{Q_{H}}{0.210\times m_{Pt}}$$

where *Q*_*H*_ is the area of hydrogen desorption from which the capacitive area is subtracted and *m*_Pt_ is the mass of Pt deposited on the electrode. Additionally, the double-layer capacitance was calculated as the slope of the capacitive current versus each scan for the different catalysts.

For the EOR, freshly deposited samples were used. The measurements were carried out in 0.5 M KOH in a nitrogen saturated atmosphere. The catalyst was first activated by cycling from 0.00 to 1.10 V vs RHE at 0.2 V/s for 50 times. Next, ethanol was introduced to a concentration of 0.5 M, and the EOR was performed from 0.00 to 1.1 V vs RHE at 0.02 V/s for 3 times. Linear Sweep Voltammetry (LSV) was performed from 0.00 to 1.1 V vs RHE at 0.05 V/s. Chronoamperometry (CA) was then performed at 0.7 V vs RHE for 500 s. To conclude, EIS was performed at 0.5 V vs RHE with an amplitude of 0.05 V from 100000 to 0.1 Hz. EIS data were fitted with the Randles-CPE equivalent model: R_s_ – (R_ct_ || CPE). R_s_ is the electrolyte resistance, R_ct_ is the charge-transfer resistance associated with the apparent kinetics of the EOR at the electrolyte/catalyst interface, Q is the constant phase element (CPE) coefficient, which describes the nonideal capacitive response of the electrochemical interface, and *n* is the CPE exponent (0 < *n* ≤ 1), which indicates the deviation from ideal capacitive behavior (*n* = 1).

To conclude, the variation of local pH at the electrolyte/catalyst interface was estimated from the HER potential shift. Inspired by Figueiredo et al., HER was performed at the Pt ring in nitrogen atmosphere by cycling the ring from −0.1 to 0.2 V vs RHE at 10 mV/s for 5 cycles.^30^ Thereafter, the same HER cycles were performed on the ring in 0.5 M ethanol, with the potential of the disk fixed at the maximum of the EOR. The variation of local pH was evaluated as follows: from the pure HER CVs, the current at 0.1 V vs RHE was selected as the reference current since at 0.1 V vs RHE, there is no interference with EOR byproducts.^30^ From the HER recorded during EOR, the potential at which the same current is obtained is considered to evaluate the shift of the potential (Δ*E* = *E* – 0.1). Therefore, from the Nernst equation, it is possible to calculate the variation of the pH as follows:

7 $$\Delta pH=-\frac{\Delta E}{0.0591}$$

## 3 Results and Discussion

### 3.1 Physiochemical Characterization

Figure 1a shows the XRD patterns of the prepared catalysts. The broad diffraction band observed at 2θ ≈ 25° in all the samples is attributed to the (002) plane of the hexagonal structure of Vulcan Carbon (VC). The diffraction peaks located at approximately 39°, 46°, and 68°, assigned to the (111), (200), and (220) crystallographic planes, respectively, confirm the presence of the face-centered cubic structure of platinum in all samples. On the other hand, no peaks referring to iron are present. Indeed, for both Pt–Fe/C catalysts, a shift to higher 2θ can be observed, suggesting that Fe was alloyed into the Pt structure,^31^ as highlighted in Figure 1b. Particularly, a higher shift is observed for the sample obtained in an alkaline medium, indicating a higher degree of incorporation of the second metal.^32^ As higher pH favors Fe reduction, the Pt–Fe/C sample prepared at pH 11.4 showed increased Fe incorporation and, consequently, a higher alloy content, consistent with Yang et al.^33^ Table 1 summarizes the XRD results for crystal size (τ), *d*-spacing, and lattice parameter (*a*) of the XRD. Regarding the Pt samples, the catalyst prepared in basic media shows a larger crystal size. Indeed, the reduction of Pt­(II) ions under alkaline conditions is slower due to the formation of hydroxide species. This can limit the nucleation rate while promoting particle growth. In addition, changes in metal–support interaction with pH result in electrostatic repulsion between Pt and VC, leading to slightly larger nanoparticle size compared to neutral conditions.^26,34^ Regarding the Pt–Fe samples, a contraction of the structure is observed in terms of τ, *d,* and *a*. A contraction of the Pt crystalline structure confirms the alloying of Fe into the Pt crystals and indicates higher Fe incorporation into the Pt lattice for Pt–Fe/C pH 11.4. Notably, such lattice contraction provides experimental evidence of a strain effect that may contribute to alter adsorption properties. Similar effects have been reported for bimetallic catalysts, where the incorporation of a second metal resulted in a lattice strain effect correlated with modifications of the Pt electronic environment, potentially affecting the adsorption energies of reaction intermediates.^35,36^ This is crucial for EOR, since the modification of the electronic structure of Pt can reduce CO adsorption and increase intermediate tolerance.^37^

**Figure 1:**
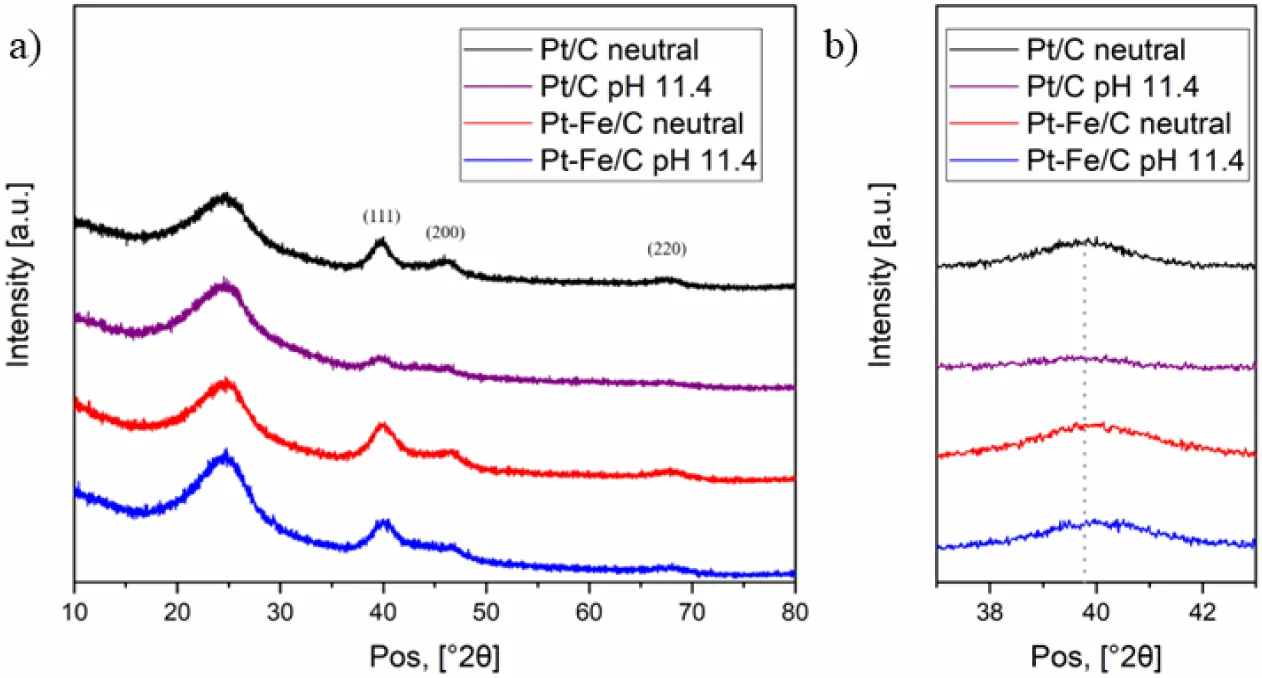
XRD patterns of Pt/C and Pt–Fe/C prepared catalysts: a) X-ray diffraction wide scan; b) zoom on the Pt(111) reflection peak.

**Table 1:** Crystal Size, d-Spacing and Lattice Parameter of the Catalysts Derived from Lorentzian Fitting of the Pt(111) Diffraction Peak

Sample	Crystal size (τ) (nm)	*d*-spacing (d) (nm)	Lattice parameter (a) (Å)
Pt/C neutral	4.55 ± 0.03	0.22620 ± 0.00002	3.9190 ± 0.0003
Pt/C pH 11.4	5.51 ± 0.02	0.2265 ± 0.0001	3.922 ± 0.002
Pt–Fe/C neutral	3.780 ± 0.001	0.2256 ± 0.0002	3.908 ± 0.004
Pt–Fe/C pH 11.4	4.31 ± 0.07	0.2252 ± 0.0001	3.901 ± 0.001

UV–vis was performed on the filtered supernatant to quantify the Pt load on the catalyst. Specifically, the Pt content on the catalyst accounted for 9.1, 9.1, 7.5, and 3.7% for Pt/C neutral, Pt/C pH 11.4, Pt–Fe/C neutral, and Pt–Fe/C pH 11.4, respectively. The equal amount of metal for pure Pt catalysts indicates that 5 h is sufficient to achieve near-complete reduction of the Pt­(II) present and reach equilibrium even for the alkaline synthesis since the Pt­(II) reduction in alkaline media is less favored kinetically.^38^ Regarding Pt–Fe samples, the simultaneous reduction of Fe and Pt ions affects the final material. During polyol synthesis, Pt is expected to nucleate first, while Fe is subsequently incorporated into the growing metallic phase. Indeed, at neutral pH a slight decrease in Pt content is observed, whereas under alkaline conditions, hydroxide ions alter precursor speciation and reduction pathways,^39^ making the reduction of Fe species kinetically more favorable. This will promote Fe incorporation while simultaneously affecting Pt deposition and reduction efficiency, resulting in a lower Pt content.

Figure 2 shows the SEM micrographs of Pt/C neutral (a), Pt/C pH 11.4 (b), Pt–Fe/C neutral (c), and Pt–Fe/C pH 11.4 (d), precisely. All of the catalysts feature a typical aggregated morphology of carbon-supported nanostructured catalysts. No large metallic segregations are observed, suggesting an overall effective deposition of the metallic phase onto the carbon matrix. Precisely, the Pt/C catalysts (Figure 2a-b) show a homogeneous morphology, characterized by smaller aggregates and a more open porous structure. In particular, Pt/C at pH 11.4 appears slightly more uniform, suggesting that alkaline conditions may influence the nucleation process during the polyol synthesis, leading to a more controlled particle distribution. In contrast, the Pt–Fe/C catalysts (Figure 2c-d) display a more compact and aggregated morphology, with the presence of larger and denser clusters with respect to Pt/C only. Indeed, the introduction of Fe can modify the reduction kinetics and mechanism, resulting in the formation of more densely packed agglomerates, as suggested by XRD as well. Additionally, Pt–Fe/C at pH 11.4 exhibits further agglomeration, compared to its neutral counterpart. This result may be attributed to higher Fe incorporation under alkaline conditions, which affects the nucleation, growth, and aggregation mechanisms during polyol synthesis. Moreover, Figure S2 shows that the EDX mappings confirm the presence of the metals in the respective catalysts. Pt appears homogeneously dispersed over the carbon matrix without the formation of large Pt-rich domains. In the case of Pt–Fe/C catalysts, Fe is uniformly distributed as well, supporting the effectiveness of the synthesis in promoting a homogeneous incorporation of Fe. Moreover, with respect to neutral Pt–Fe, by increasing the pH, the qualitative ratio of Fe:Pt increases from 0.037 to 0.056, supporting the hypothesis of further Fe incorporation under alkaline conditions, consistent with the larger XRD peak shift and lattice contraction. The metal contents and ratios were confirmed by ICP-MS.

**Figure 2:**
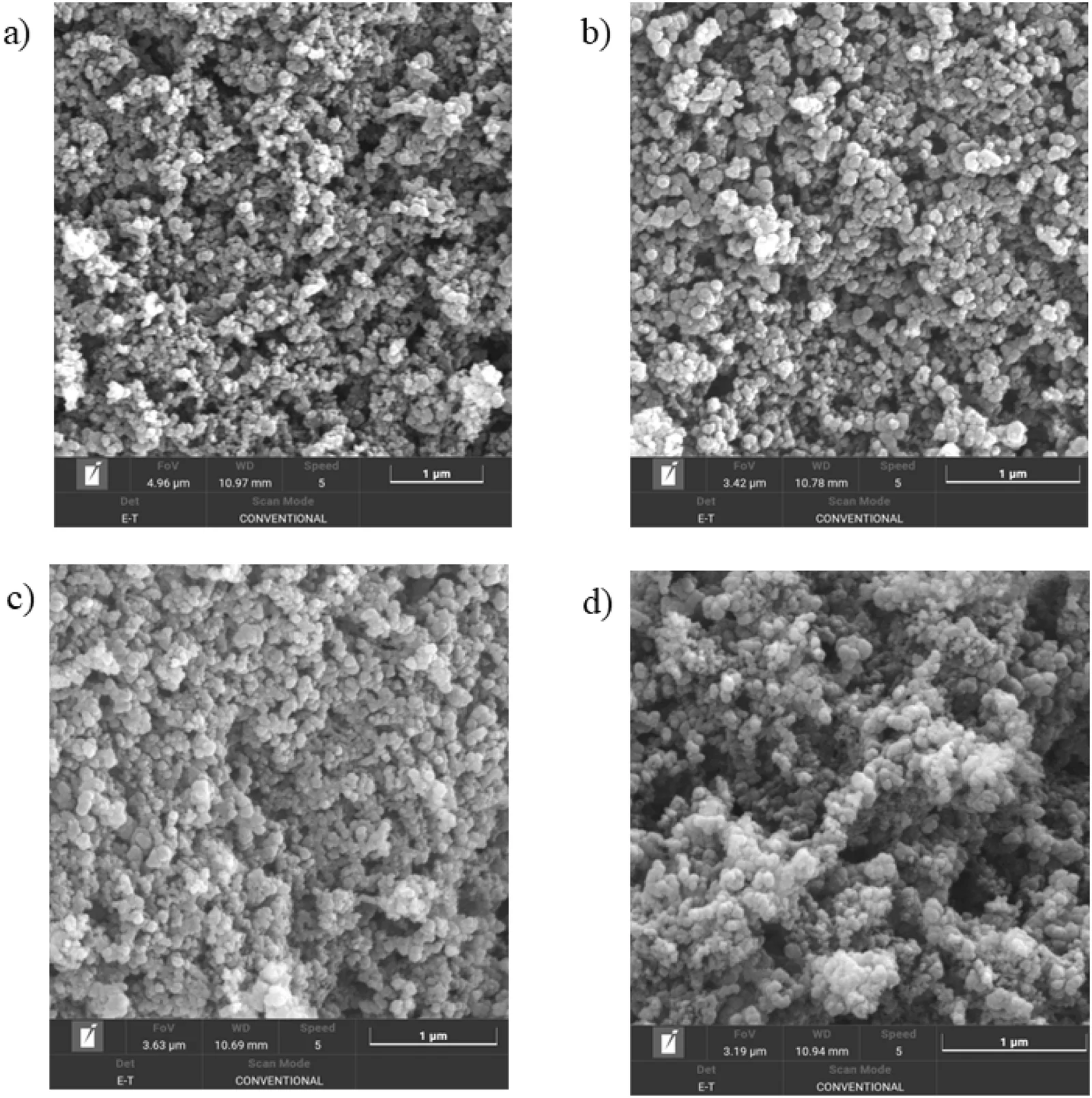
SEM-EDX of Pt/C neutral (a), Pt/C pH 11.4 (b), Pt–Fe/C neutral (c), and Pt–Fe/C pH 11.4 (d).

### 3.2 Electrochemical Characterization

Figure 3a shows the cyclic voltammograms of the catalysts prepared. The CVs were recorded in a nitrogen atmosphere and 0.1 M HClO_4_ as an electrolyte solution at a scan rate of 50 mV/s. The total amount of catalyst was kept constant; therefore, the actual Pt content varied according to the load of Pt, as reported in Table S1. For all catalysts, it is possible to notice the hydrogen adsorption/desorption peak characteristics of polycrystalline platinum (0.05–0.38 V vs RHE). The ECSA was calculated from the area of hydrogen desorption peaks between 0.03 and 0.38 V vs RHE after eliminating the contribution of the double layer. The values are 41.9, 38.5, 42.9, and 101.3 m^2^/g_Pt_ for Pt/C neutral, Pt/C pH 11.4, Pt–Fe/C neutral, and Pt–Fe/C pH 11.4, respectively. The highest ECSA of the latter can provide more active sites for the EOR and therefore enhance its catalytic activity. It is possible to notice that the peak of hydrogen desorption at ca. 0.07 V vs RHE of Pt–Fe/C pH 11.4 is wider and slightly shifted relative to the other samples, evidencing the effects on Pt surface sites by Fe incorporation. This behavior suggests a modification of the electronic structure of Pt induced by Fe loading. Indeed, strain and ligand effects lead to electronic perturbation on Pt surface sites, inducing a modification of hydrogen adsorption energy, as indicated by the positive shift of the hydrogen desorption peak.^40,41^ Similar broadening was observed by Qu et al., which produced a PtFe dendritic nanowire alloy.^42^ The introduction of Fe modulated the electronic structure of Pt, resulting from synergy between ligand and strain effects, leading to enhanced catalytic activity. Figure 3b shows the non-Faradaic current vs the scan rate in the range of 0.5–0.6 V vs RHE. The slope of the plot is the double-layer capacitance (*C*_*dl*_), which is calculated to be equal to 145, 62, 111, and 295 F/g for Pt/C neutral, Pt/C pH 11.4, Pt–Fe/C neutral, and Pt–Fe/C pH 11.4, respectively. These results correspond to an increased interfacial area, which leads to more exposed active sites and therefore a higher electrochemically active surface area of Pt–Fe/C pH 11.4 due to the alloying of Fe.^43^

**Figure 3:**
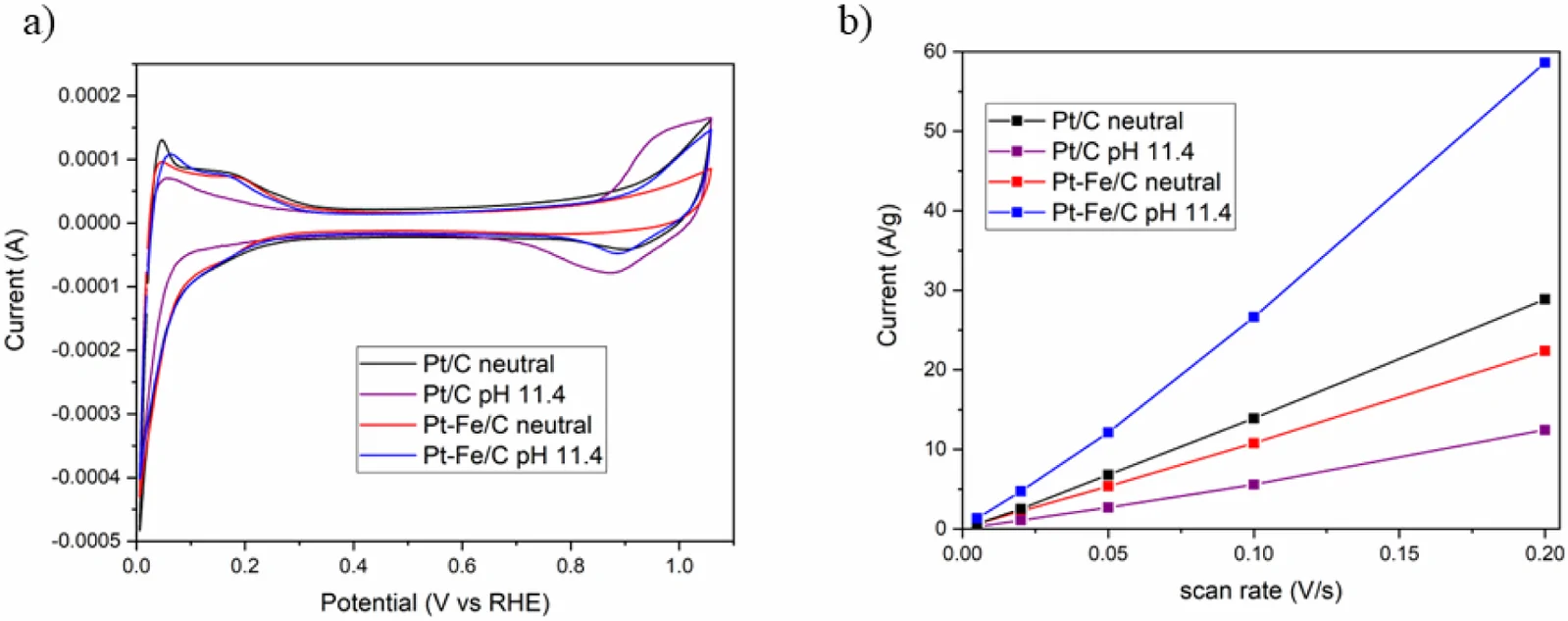
Cyclic voltammetry at 50 mV/s (a) and capacitive current vs scan rate (b) in N_2_ atmosphere and 0.1 M HClO_4_ solution of Pt/C neutral, Pt/C pH 11.4, Pt–Fe/C neutral, and Pt–Fe/C pH 11.4.

Figure 4 displays the EOR activities of the prepared catalysts at 20 mV/s. The only Pt catalysts (Figure 4a and Figure 4b) show similar behavior, with a maximum oxidation current of 250 A/g, consistent with their comparable crystal size and ECSA. By alloying Fe into the catalyst, the peak current increases further. Indeed, Pt–Fe/C neutral (Figure 4c) reaches a maximum of ca. 530 A/g, while Pt–Fe/C pH 11.4 (Figure 4d) reaches a maximum of ca. 850 A/g_Pt_. The higher activity of the latter can be due to a higher Fe alloyed thanks to the alkaline conditions. Indeed, despite Pt–Fe/C neutral having higher Pt content with respect to Pt–Fe/C pH 11.4, the activity is found to be lower: higher Pt content is not sufficient for reaching higher current density. The role of Fe is crucial since it promotes the adsorption and therefore the reaction of OH^–^ with adsorbed intermediates and CO to produce CO_2_ and H_2_O.^33^ This is confirmed by the *I*_*f*_ /*I*_*b*_ ratio. *I*_*f*_ is the peak current of the forward scan associated with the direct oxidation of ethanol, while *I*_*b*_ is the peak of the backward/reverse scan associated with the oxidation of partially oxidized intermediates accumulated during the forward scan. A higher ratio qualitatively indicates an enhanced tolerance toward poisoning by intermediates and an efficient removal of the species. Indeed, Pt–Fe/C neutral features a ratio equal to ca. 1.6, while Pt–Fe/C pH 11.4 has a value of ca. 1.9. Therefore, the increased ratio confirms that the higher alloying of Fe promotes the adsorption of OH^–^ at lower potentials, thus promoting the oxidative removal of CO-like intermediates.^44^ This results in a larger number of active Pt sites available during the forward scan, leading to an overall improvement in the EOR. Moreover, the variation of crystal sites induced by Fe alloying, as proven by the positive shift of the hydrogen desorption peak, can modify adsorption energies and the bonding strength of the carbonaceous intermediates, therefore resulting in higher EOR activity.^45^

**Figure 4:**
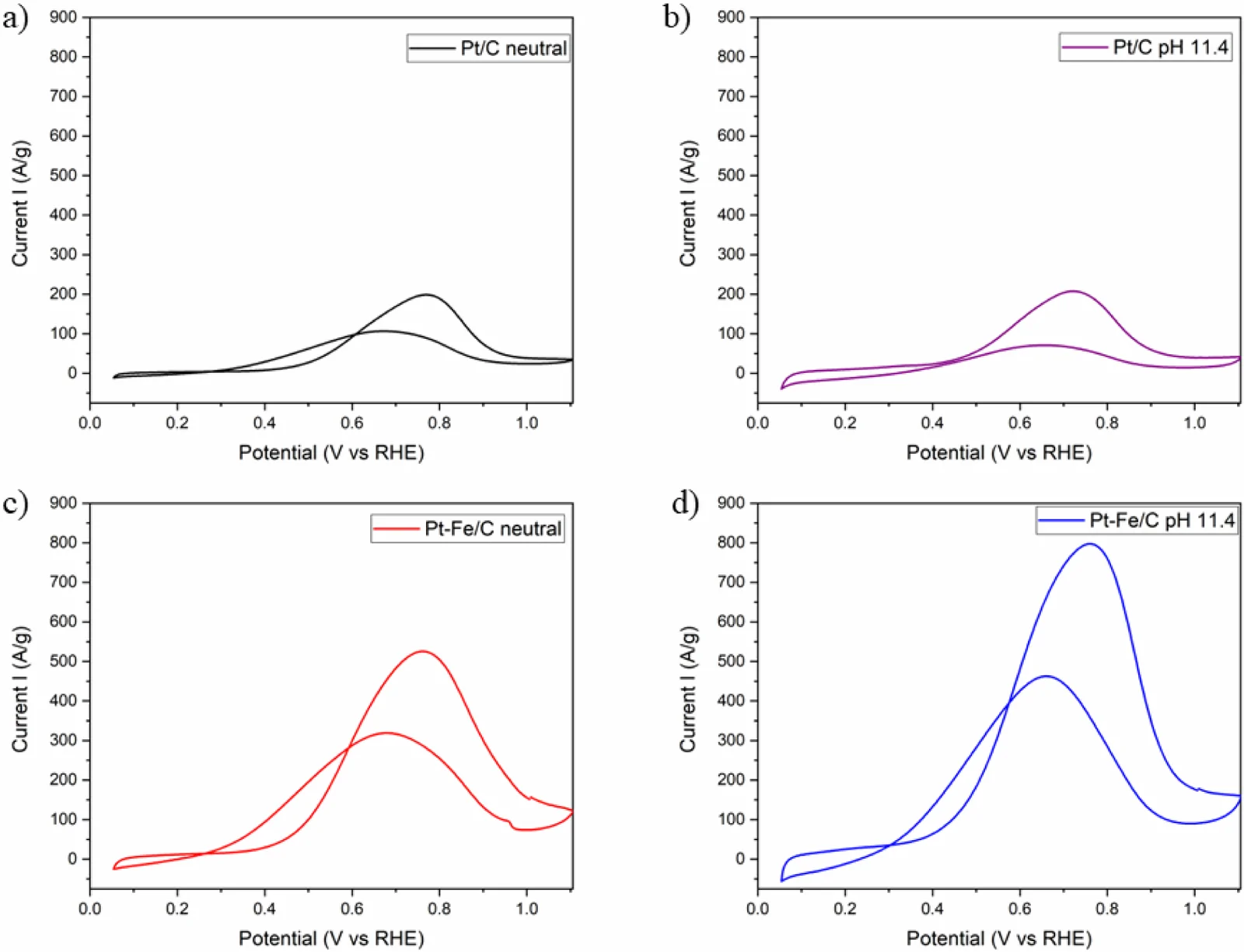
CVs in 0.5 M ethanol in a nitrogen atmosphere and 0.5 M KOH solution at 20 mV/s of Pt/C neutral (a), Pt/C pH 11.4 (b), Pt–Fe/C neutral (c), and Pt–Fe/C pH 11.4 (d).

Figure 5a displays the LSV curves of the investigated materials. From these plots, the onset potentials were estimated to be 0.479, 0.497, 0.503, and 0.517 V vs RHE for Pt–Fe/C pH 11.4, Pt–Fe/C neutral, Pt/C pH 11.4, and Pt/C neutral, respectively. The negative-shift of the onset potential for Fe-alloyed samples indicates enhanced EOR kinetics at lower potentials, with outstanding activity for Pt–Fe/C pH 11.4. Indeed, this behavior can be attributed to the bifunctional mechanism where Fe sites promote the adsorption of hydroxyl species at lower potentials, thereby promoting the reaction.^46^ Tafel analysis was performed in the 0.50–0.60 V vs RHE region. The Pt–Fe catalysts exhibited lower Tafel slopes than the corresponding monometallic Pt samples, with values of 160 and 150 mV/dec for Pt–Fe/C neutral and Pt–Fe/C pH 11.4, compared with 180 and 229 mV/dec for Pt/C neutral and Pt/C pH 11.4. The lower slope observed for Pt–Fe/C pH 11.4 is consistent with more favorable apparent EOR kinetics. To further evaluate steady-state EOR performance, chronoamperometry tests at steady-state potential were carried out. Figure 5b displays chronoamperometry plots in 0.5 M ethanol and 0.5 M KOH electrolyte solution at 0.7 V vs RHE. All catalysts show a current decay over time, suggesting the production of adsorbed reaction intermediates during operation. At longer times, the current decay becomes considerably slower, indicating the establishment of a quasi-steady state resulting from the balance between ethanol oxidation, formation of adsorbed intermediates, and their oxidative removal. Among all the catalysts, Pt–Fe/C pH 11.4 shows the highest current, followed by Pt–Fe/C neutral, while both Pt/C catalysts exhibit lower residual currents. This behavior suggests that Fe incorporation improves not only EOR activity but also the ability of the catalyst to sustain EOR under prolonged polarization times. The higher residual current of Pt–Fe/C pH 11.4 is therefore consistent with a greater tolerance toward surface deactivation by adsorbed intermediates, possibly promoted by the bifunctional contribution of Fe-containing sites to the oxidative removal of poisoning species. These results agree with the CV and LSV results. Moreover, EIS was performed for all the samples to evaluate the EOR dynamics. All plots (Figure 5c) show a semicircular arc that corresponds to the charge transfer resistance (R_ct_) involved with the EOR. The EIS data have been fitted with the the Randles-CPE model, and the parameters are reported in Table S2. Particularly, R_ct_ decreases in the following order:

**Figure 5:**
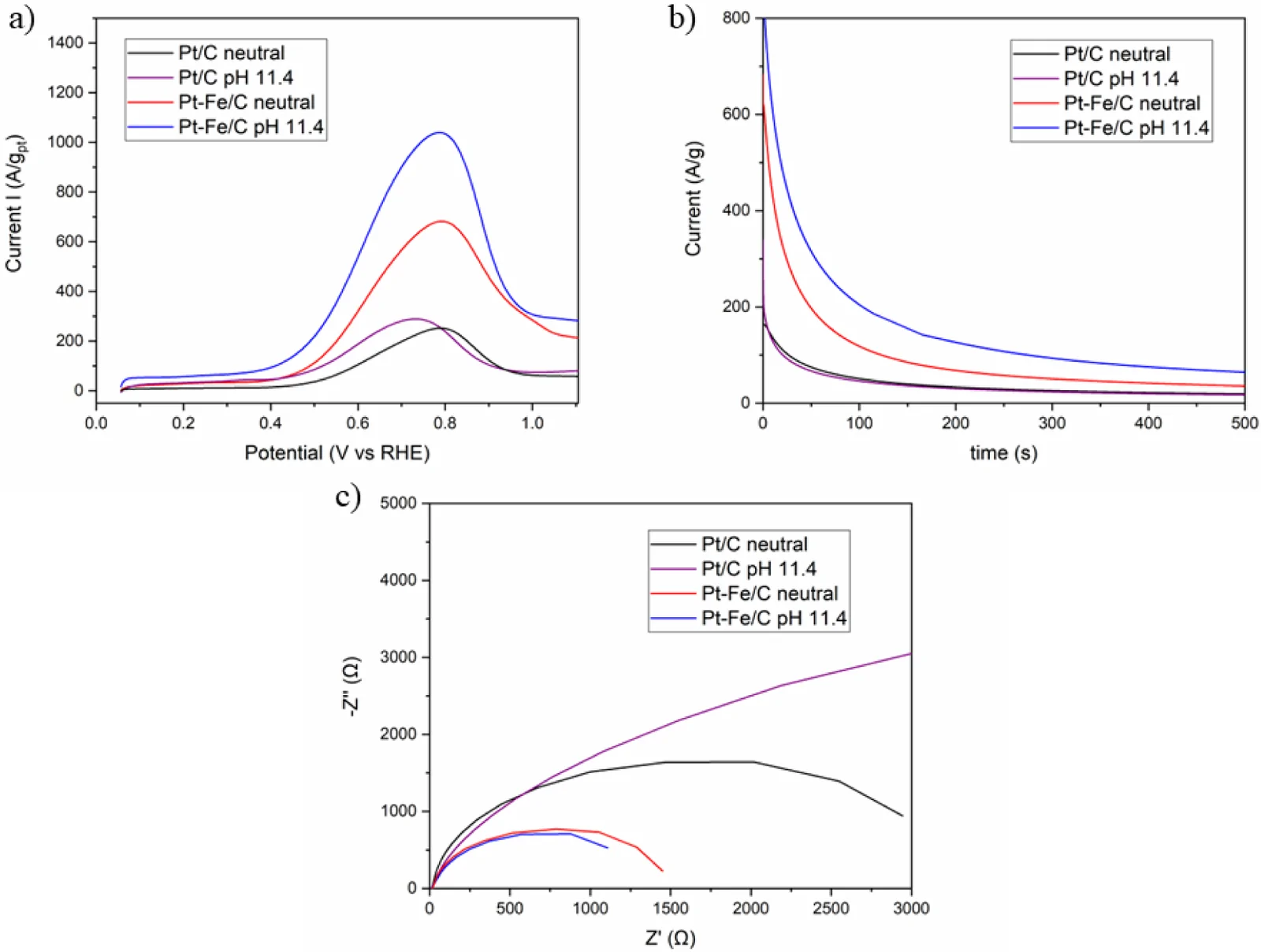
LSV curves at 50 mV/s in a nitrogen atmosphere (a), chronoamperometry at 0.7 V vs RHE (b), and EIS at 0.5 V vs RHE (c) of Pt/C neutral, Pt/C pH 11.4, Pt–Fe/C neutral, and Pt–Fe/C pH 11.4, all in a 0.5 M KOH electrolyte solution and 0.5 M ethanol.

Pt/C pH 11.4 (10086 Ω) > Pt/C pH 11.4 (3218 Ω) > Pt–Fe/C neutral (1445 Ω) > Pt–Fe/C pH 11.4 (1220 Ω).

A similar trend has been reported in the literature, where a lower charge transfer resistance (R_ct_) is observed for a more active electrocatalyst, indicating enhanced reaction kinetics.^47^ Therefore, the EIS results confirm the CV and LSV results, where Pt–Fe/C at pH 11.4 is the most active catalyst despite the lower Pt content, highlighting the beneficial role of Fe alloying.

To evaluate the effect of Fe incorporation on hydroxyl species adsorption on the working electrode, HER measurements as a pH probe during EOR were performed. Pt–Fe/C at pH 11.4 was selected as the best catalyst and compared with commercial Pt/C. Figure 6a and Figure 6b show the HER polarization curves for Pt/C and Pt–Fe/C at pH 11.4, respectively, without and with 0.5 M ethanol. In both cases, the HER curves obtained under reactive conditions are shifted in comparison with the reference HER curves, suggesting that EOR alters the local interfacial microenvironment. The potential shift at the ring, ΔE, was larger for Pt–Fe at pH 11.4 with respect to Pt/C (0.066 V vs 0.036 V, respectively), which corresponds to a variation of pH equal to 1.11 and 0.69, respectively. This result suggests a stronger perturbation of the interfacial hydroxide environment for Pt–Fe/C at pH 11.4, being consistent with a greater involvement of hydroxyl species during EOR at the disk. Such behavior may be associated with the oxyphilic character of Fe-containing sites, which promotes the formation of oxygenated surface species involved in the oxidative removal of adsorbed intermediates.

**Figure 6:**
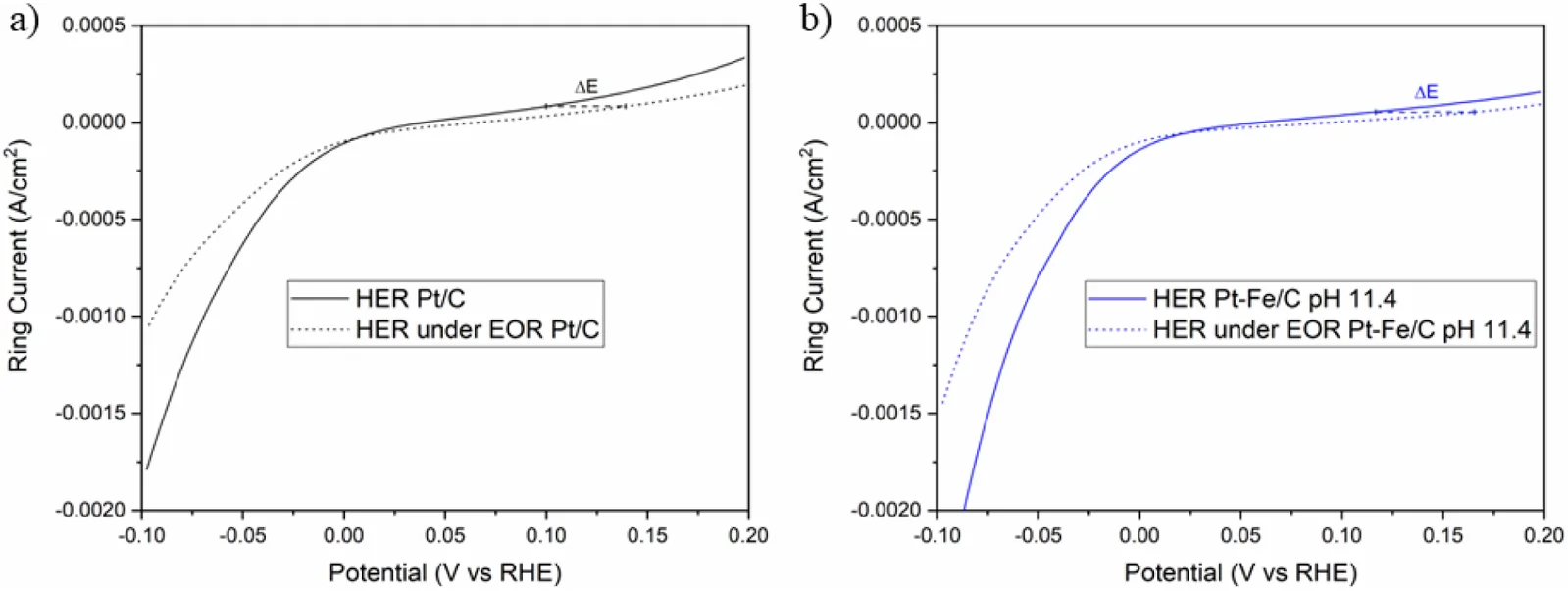
HER polarization curves of Pt/C (a) and Pt–Fe/C pH 11.4 (b). Solid lines represent the HER recorded at the Pt ring in a nitrogen atmosphere under nonreactive conditions, while dashed lines correspond to the HER conducted under EOR conditions, with the disk held at the EOR peak potential.

To conclude, Figure 7a compares Pt–Fe/C at pH 11.4 with commercial Pt/C in terms of EOR performance normalized for grams of platinum (Figure 7a). It is possible to appreciate the higher activity on the catalyst in terms of a maximum current of ca. 850 A/g_Pt_ compared to commercial Pt/C that reaches only 600 A/g_Pt_ at 20 mV/s. The enhanced activity of Pt–Fe/C at pH 11.4 can also be correlated with the lattice contraction observed by XRD, which suggests strain effects and potentially leads to a modification of the electronic properties of Pt. This electronic modification can weaken the adsorption strength of poisoning intermediates, further contributing to an improved catalytic performance.

**Figure 7:**
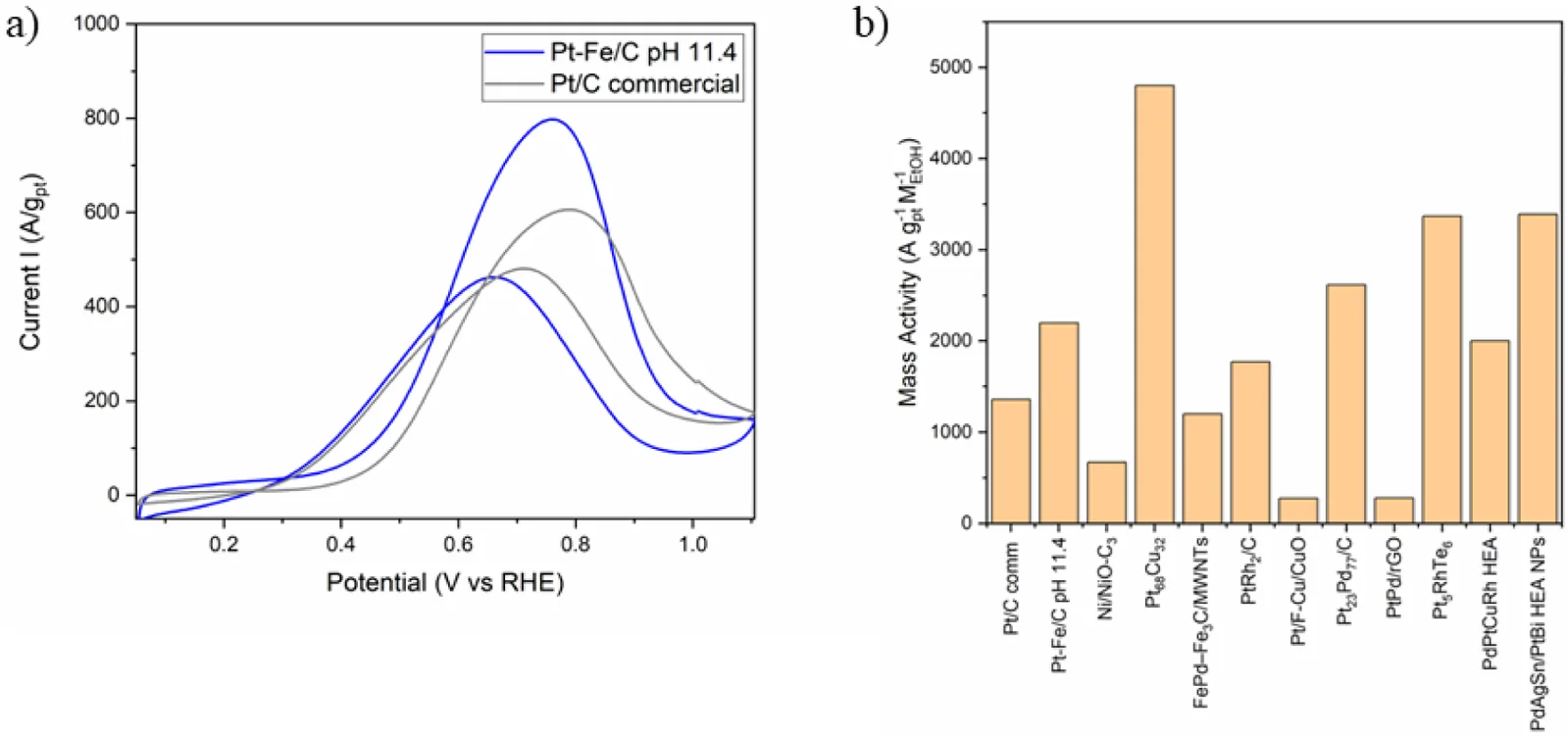
(a) CVs in 0.5 M ethanol in a nitrogen atmosphere and 0.5 M KOH solution at 20 mV/s of Pt/C commercial and Pt–Fe/C pH 11.4; (b) mass activity of different catalysts in the literature (references in Table 2), normalized for concentration of ethanol (M).

The forward oxidation peak of Pt–Fe/C occurs at a lower potential than that of commercial Pt/C (0.759 and 0.791 V vs RHE, respectively), indicating a more favorable EOR voltammetric response under the conditions investigated. This behavior may be associated with electronic and bifunctional effects arising from the presence of Fe. The *I*_*f*_/*I*_*b*_ ratios are 1.9 and 1.2 for Pt–Fe/C and Pt/C, respectively. While this ratio has been used as a qualitative indicator of tolerance to adsorbed intermediates, the backward peak is also influenced by the upper potential limit and surface oxide formation and reduction. Therefore, the ratio is reported here as a comparative descriptor of the voltammetric responses measured under the identical conditions.

Figure 7b compares different Pt-based catalysts and alloys in terms of mass activity normalized for the concentration of ethanol (M). This normalization was introduced in order to facilitate comparisons between catalysts in the literature tested under different experimental conditions, such as different ethanol concentrations, which can strongly influence the measured current density. However, the dependence of the EOR current on ethanol concentration is not necessarily linear due to the multistep nature of the process, which can modify the apparent reaction order. Therefore, normalization by ethanol concentration should be regarded as a pragmatic comparative approach rather than a rigorous kinetic correction.^48^ It is possible to notice that our catalyst features comparable activity with the catalyst obtained or higher. In this case, the improved catalytic performance of Pt–Fe/C at pH 11.4 can be attributed to the synergistic combination of increased active surface area, enhanced intrinsic activity, and improved tolerance toward poisoning intermediates, resulting from both electronic and bifunctional effects induced by Fe incorporation. Table 2 compares these catalysts in terms of mass activity and peak potential. It is interesting to note the lower potential of the peak of Pt–Fe/C at pH 11.4 in comparison with other catalysts. The bifunctional role is manifested as follows:

**Table 2:** Comparison of EOR Performance between Different Catalysts

Electrocatalyst	Synthesis	Electrolyte/Ethanol molarity	Peak Potential (V vs RHE)	Peak current at 50 mV/s (A/g_Pt_)	ref.
Pt–Fe/C pH 11.4	Polyol method without further thermal treatment	0.5 KOH/0.5 Ethanol	0.76	1100	This work
Commercial Pt/C	-	0.5 KOH/0.5 Ethanol	0.79	680	This work
Pt_68_Cu_32_ nanoalloy	One-pot route	0.5 KOH/0.5 Ethanol	0.95	2400	^49^
FePd–Fe_3_C/MWNTs	One-pot reflux solution with DMF at 160 °C	1 KOH/1 Ethanol	0.77	1200	^50^
PtRh_2_/C	Microwave-assisted polyol	1 KOH/1 Ethanol	0.824	1770	^51^
Ni/NiO-C_3_	Glucose impregnation, high temperature pyrolysis and air calcination at 400 °C	0.5 NaOH/0.5 Ethanol	1.75	335	^52^
Pt/F–Cu/CuO	Aqueous ammonia solvothermal + KBH4 reduction	1 KOH/1 Ethanol	0.87	275	^53^
PtPd_3_/Mn_3_O_4_–CNT	Modified polyol method	0.5 H_2_SO_4_/0.5 Ethanol	0.5	550	^54^
Pt_23_Pd_77_/C	Formic acid reduction method	1 KOH/1 Ethanol	0.85	2616	^44^
Dendritic PtPd/rGO	One-pot route with soft template	1 KOH/1 Ethanol	0.97	280	^55^
Pt_5_RhTe_6_ nanotubes	Solvothermal method and modified polyol method with Te reduction	1 KOH/1 Ethanol	0.88	3370	^56^
PdPtCuRh HEA	Two-steps synthesis with acetic acid and EG	1 KOH/1 Ethanol	0.82	2000	^57^
PdAgSn/PtBi HEA NPs	Wet chemical synthesis at 160 °C	1 KOH/1 Ethanol	0.9	3390	^58^

- the activation of the adsorption of OH^–^ at lower potential to facilitate the oxidation of intermediates;

- strain effects that modulate the electronic properties of Pt and therefore weakened the Pt-CO bond, resulting in increased resistance to pollutants.

## 4 Conclusions

In this work, Pt/C and Pt–Fe/C catalysts were successfully synthesized via a one-pot polyol method, and the effect of pH on alloy formation and EOR performance was investigated. The results demonstrate that the pH of the synthesis medium plays a key role in controlling Fe incorporation and, consequently, the catalytic behavior of the materials. Indeed, the catalyst prepared under alkaline conditions (Pt–Fe/C, pH 11.4) showed enhanced performance compared to the other samples, despite its lower Pt content. In particular, with respect to neat Pt/C and Pt–Fe/C synthesized at neutral pH, it exhibited the highest electrochemical surface area (above 100 m^2^/g_Pt_) and delivered substantially higher oxidation currents, together with a clear shift of the onset potential toward lower values. The improved activity can be attributed to both structural and electronic effects induced by Fe alloying. The lattice contraction observed by XRD suggests that strain effects can modify the electronic structure of Pt and weaken the adsorption strength of poisoning intermediates. At the same time, Fe contributes through a bifunctional mechanism by facilitating OH^–^ adsorption at lower potentials, thus promoting the oxidation of carbonaceous species accumulated during the reaction. Overall, this study demonstrates that tuning the pH during polyol synthesis is an effective strategy to tailor Pt-based alloy catalysts.

## Supplementary Material



## Notes

UV–vis calibration curve used to determine the Pt content (**Figure S1**); SEM-EDX images of Pt/C neutral, Pt/C pH 11.4, Pt–Fe/C neutral, and Pt–Fe/C pH 11.4 (**Figures S2a–d)**; cyclic voltammograms recorded at scan rates of 0.2, 0.1, 0.05, 0.02, and 0.005 V/s and used to evaluate the double-layer capacitance (c_dl_) provided for Pt–Fe/C pH 11.4 (**Figure S3**), Pt/C pH 11.4 (**Figure S4**), Pt–Fe/C neutral (**Figure S5**), and Pt/C neutral (**Figure S6**); **Table S1** reports the Pt weight percentage of each catalyst together with the corresponding Pt loading used for electrochemical measurements; **Table S2** summarizes the EIS fitting parameters obtained using the modified Randles model for the investigated catalysts during the ethanol oxidation reaction­(EOR) (PDF)
