# Supplementary material for: One-Pot Synthesis of Pt–Fe for EOR: Effect of pH
Source: ACS Omega. 2026 Oct 19;11(43):67679–89. doi: 10.1021/acsomega.6c09204 (PMC13737973; doi:10.1021/acsomega.6c09204)
Supplement: Supplementary file 1 [file ao6c09204_si_001.pdf]

One-pot synthesis of Pt-Fe for EOR: effect of pH

Marco Antonio Di Francia, Maria Portarapillo, Antonella Gianna Tesone, Almerinda Di Benedetto, Giuseppina Luciani*

Department of Chemical, Materials and Production Engineering, University of Naples Federico II, P.le Tecchio 80, 80125, Naples, Italy

Corresponding author: *marcoantonio.difrancia@unina.it

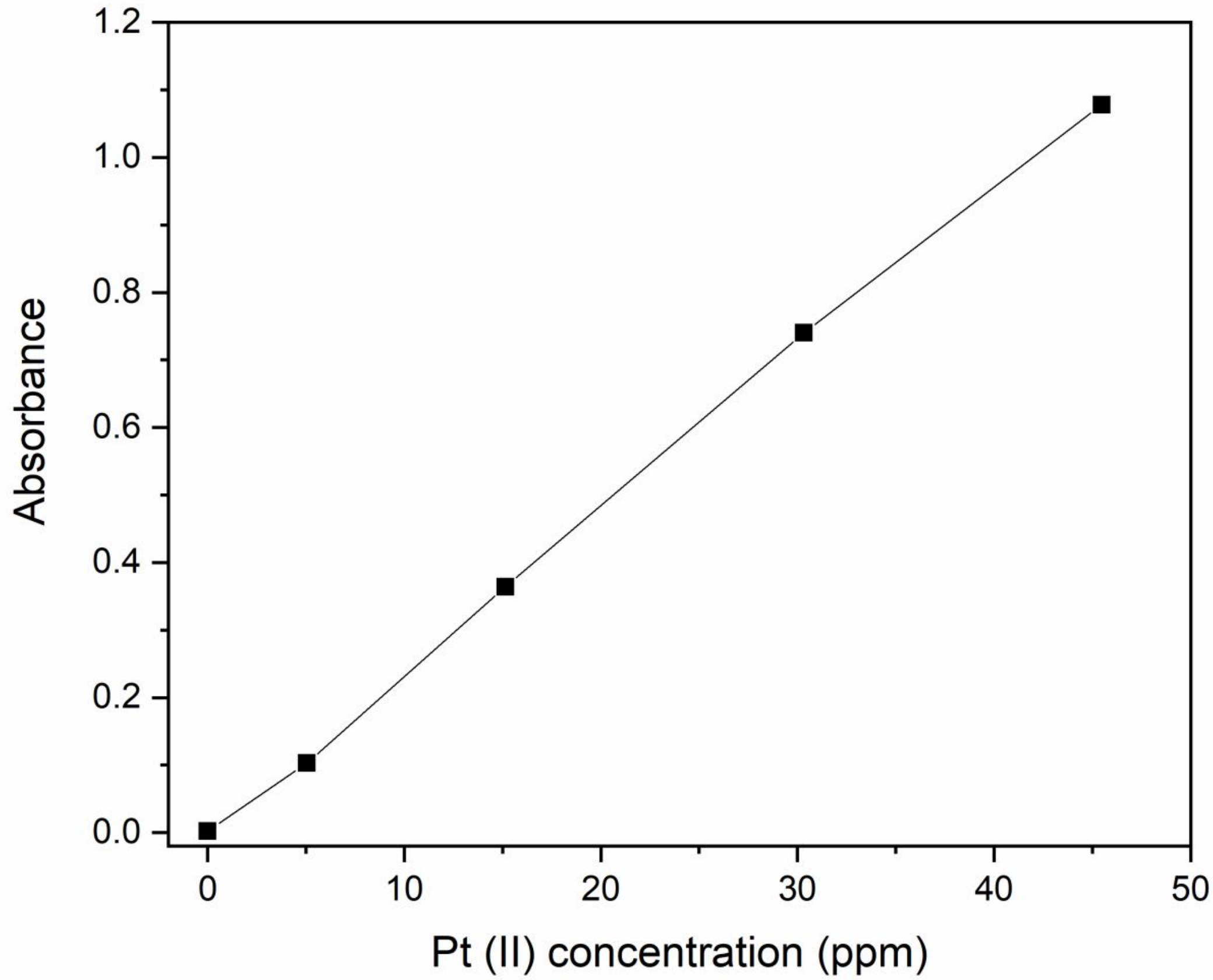

Figure S 1 - Uv-Vis calibration curve

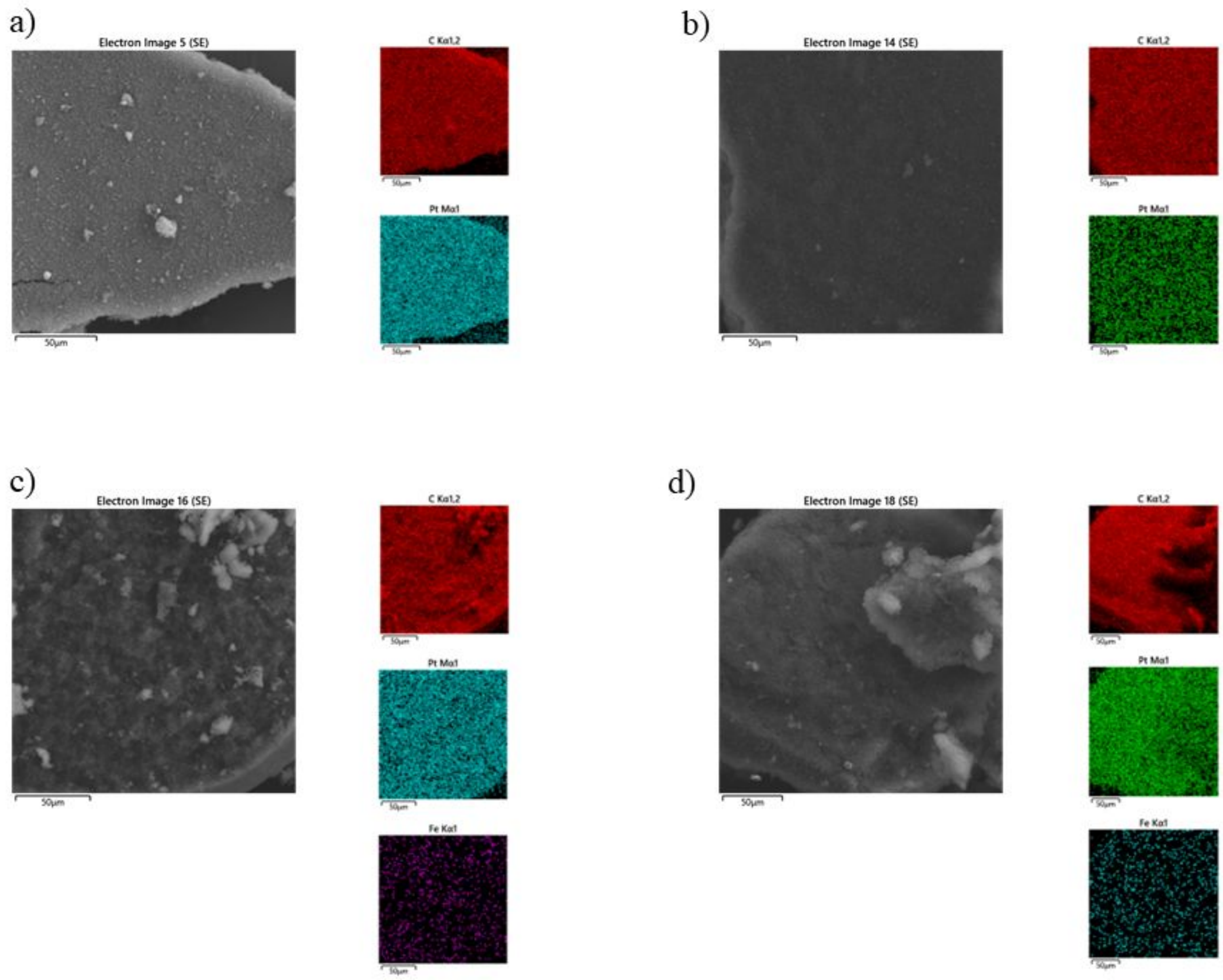

Figure S 2 – SEM-EDX of (a) Pt/C neutral, (b) Pt/C pH 11.4, (c) Pt-Fe/C neutral, (d) Pt-Fe/C pH 11.4.

Table S 1 - %Pt wt concentration and catalyst ink loading of the analyzed samples

Samples	%Pt cat	Pt loading ink
Pt/C neutral	9.1	0.017
Pt/C pH 11.4	9.1	0.017
Pt-Fe/C neutral	7.5	0.014
Pt-Fe/C pH 11.4	3.7	0.0069

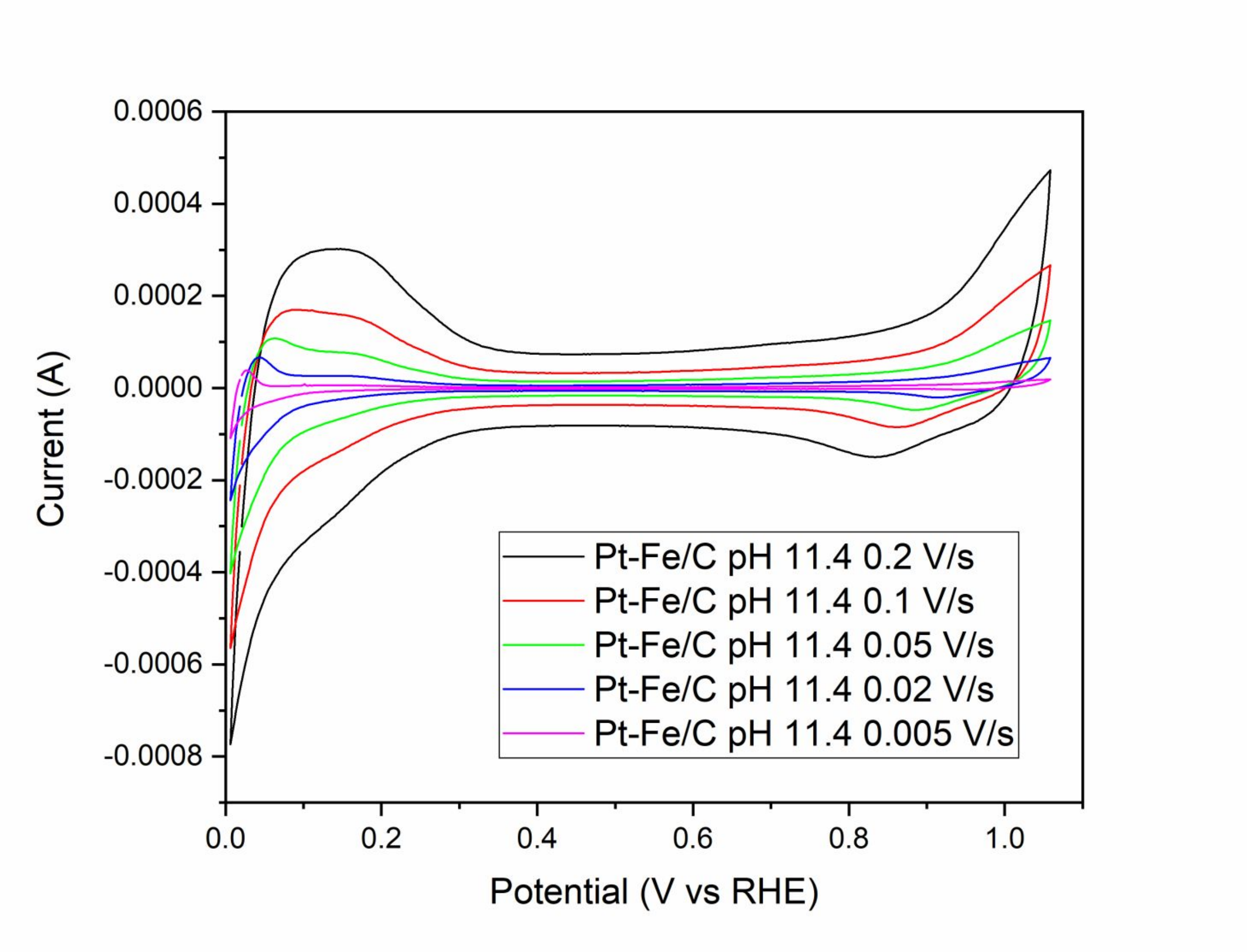

Figure S 3 – Cyclic voltammetry of Pt-Fe/C pH 11.4 at 0.2, 0.1, 0.05, 0.02 and 0.005 V/s used to evaluate c_{dl}

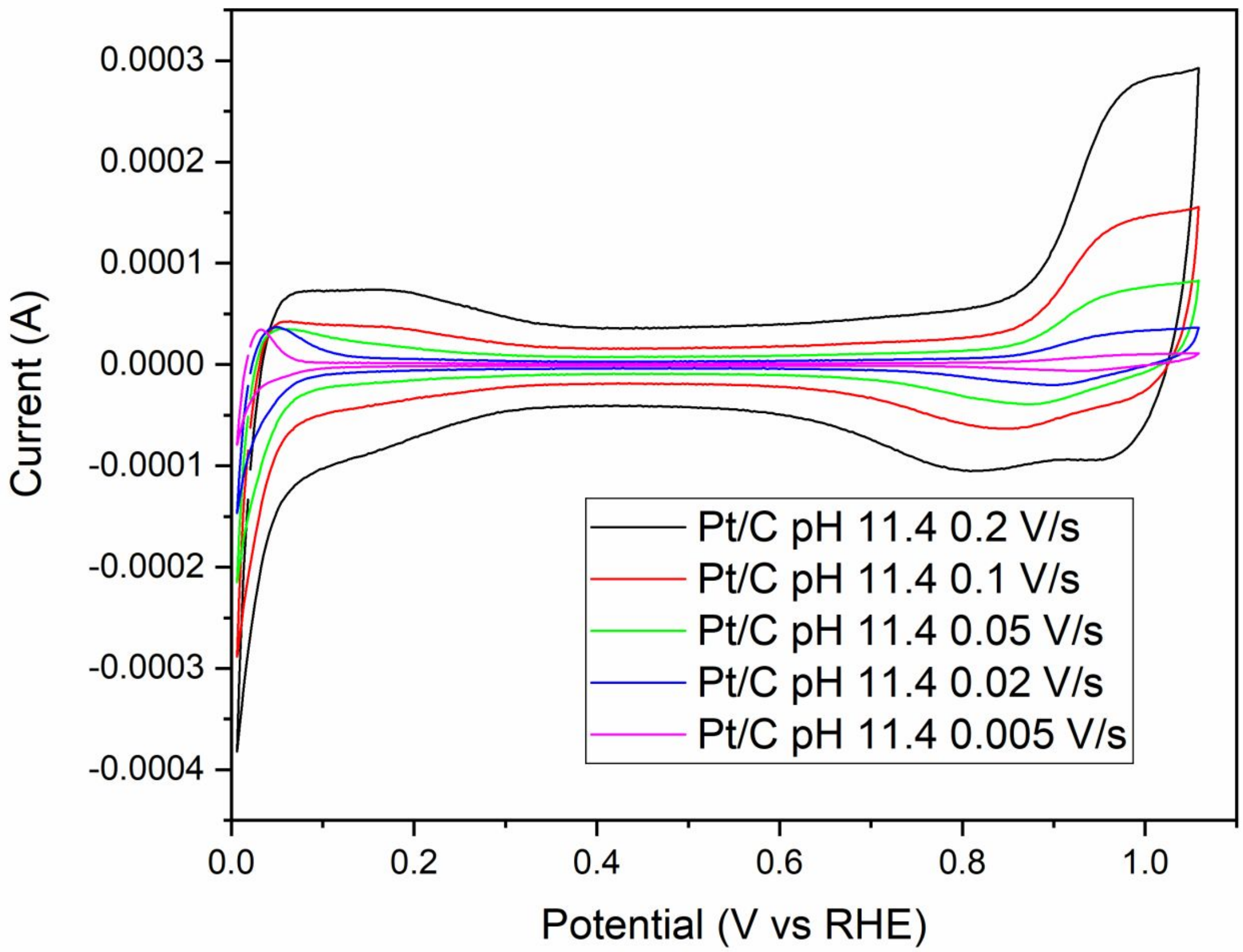

Figure S 4 – Cyclic voltammetry of Pt/C pH 11.4 at 0.2, 0.1, 0.05, 0.02 and 0.005 V/s used to evaluate c_{dl}

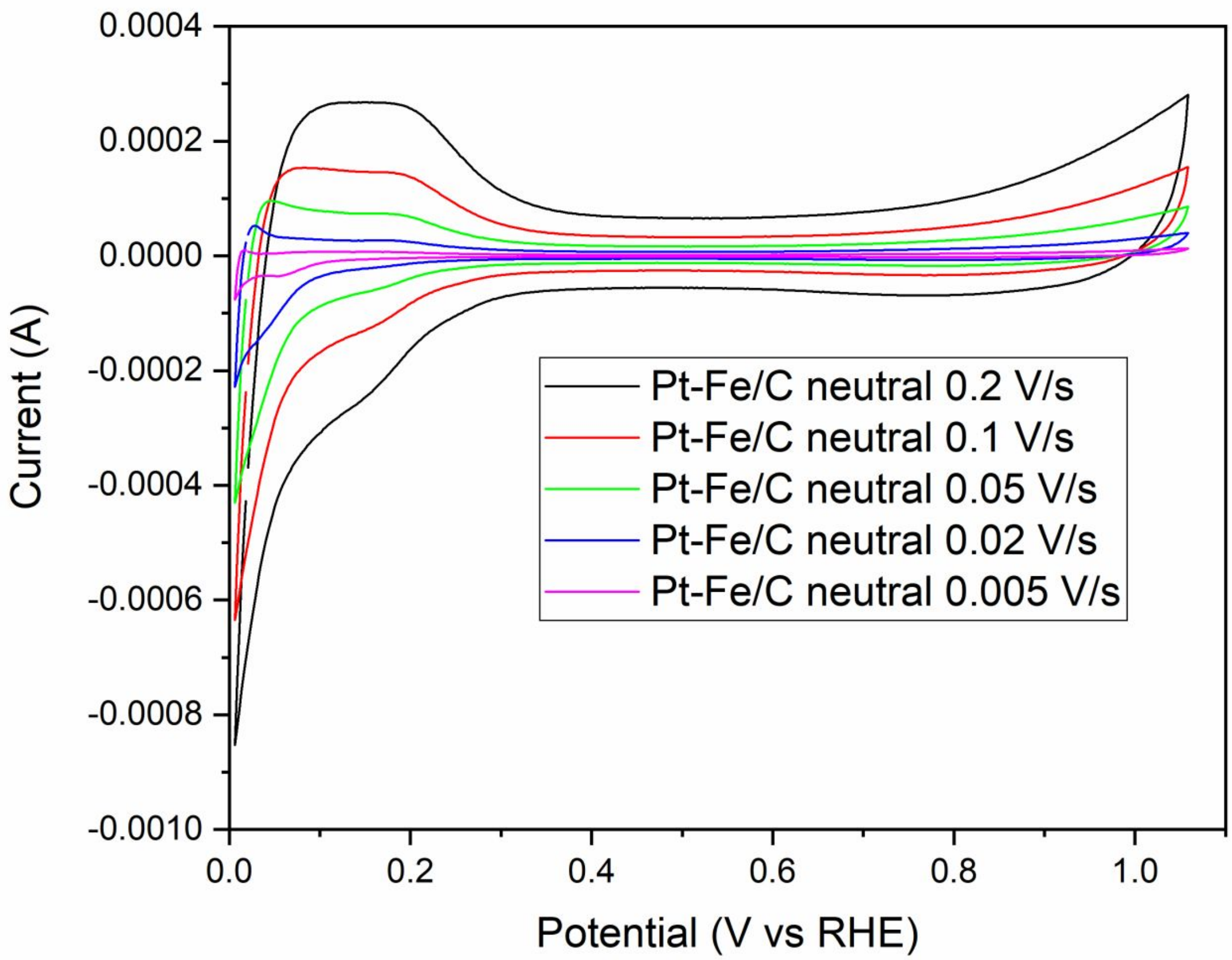

Figure S 5 - Cyclic voltammetry of Pt-Fe/C neutral at 0.2, 0.1, 0.05, 0.02 and 0.005 V/s used to evaluate c_{dl}

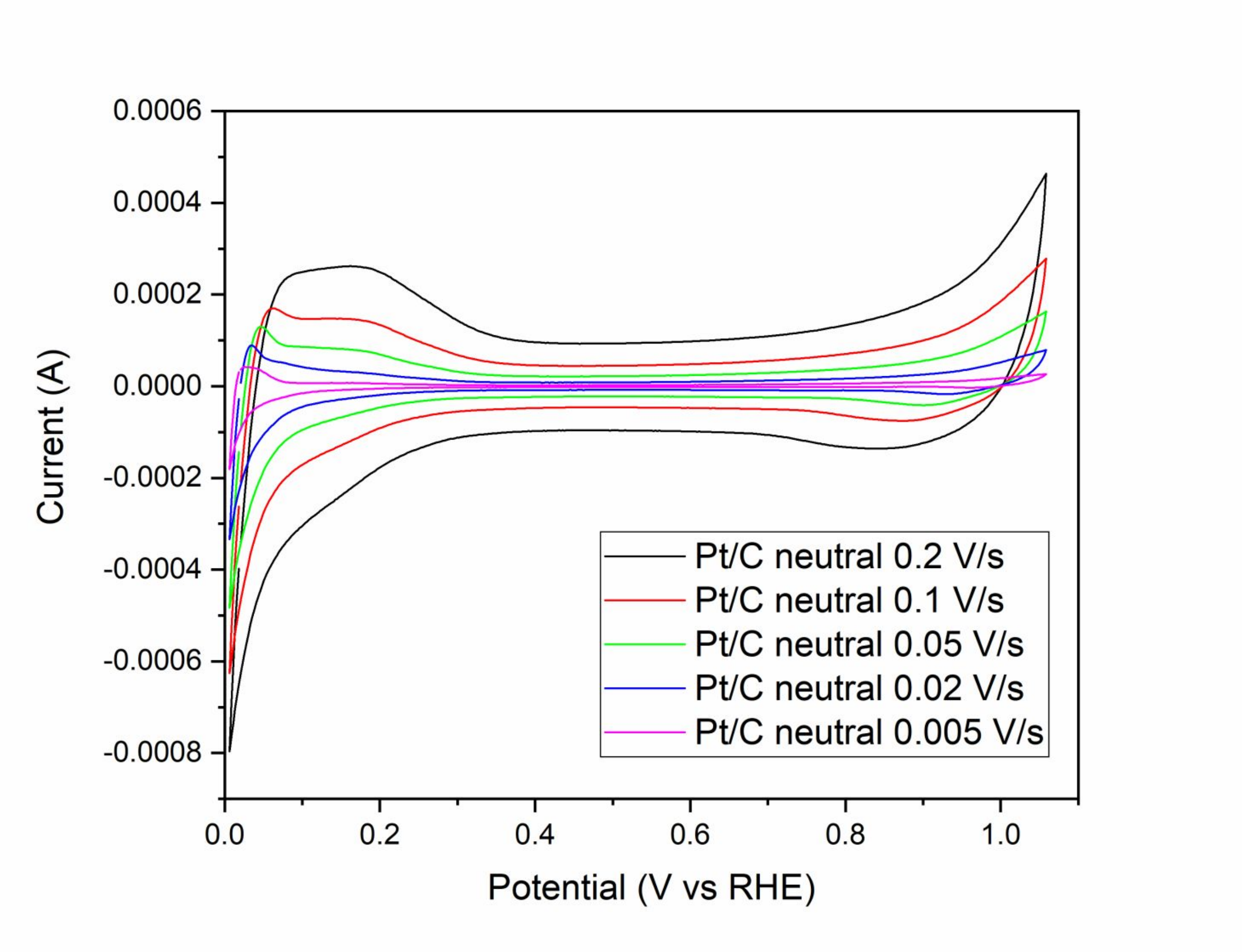

Figure S 6 - Cyclic voltammetry of Pt/C neutral at 0.2, 0.1, 0.05, 0.02 and 0.005 V/s used to evaluate c_{dl}

Table S2 – EIS fitted parameters with modified Randle model.

Sample	R_s	R_{ct}	Q	n	c_{dl}
Pt/C neutral	19	3218	0.26	1	0.26
Pt/C pH 11.4	25.4	10086	0.21	1	0.21
Pt-Fe/C neutral	19.37	1445	0.29	1	0.29
Pt-Fe/C pH 11.4	18.2	1220	0.29	1	0.29
